# Supplementary material for: Multi-Omic Temporal Landscape of Plasma and Synovial Fluid-Derived Extracellular Vesicles Using an Experimental Model of Equine Osteoarthritis
Source: Int J Mol Sci. 2023 Oct 4;24(19):14888. doi: 10.3390/ijms241914888 (PMC10573509; doi:10.3390/ijms241914888)
Supplement: Supplementary file 1 [file ijms-24-14888-s001.zip › Supplementary Figure 1 EV diagram.docx]

**Supplementary Figure 1.** Overview of the biological role of EV cargo


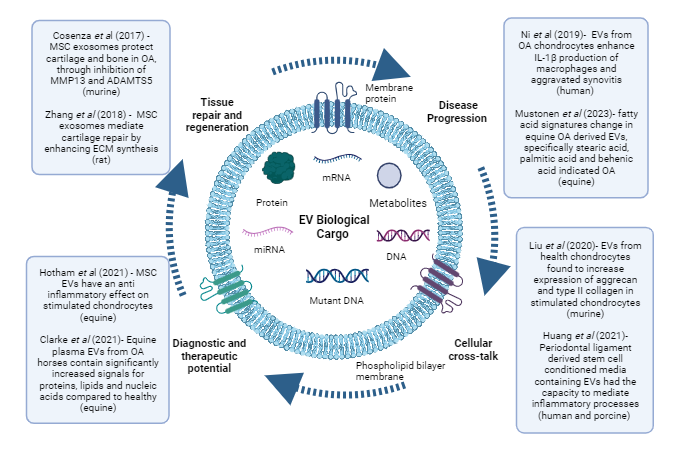


[1-8]

**References**

1. Huang, C.Y., et al., *Anti-Inflammatory Effects of Conditioned Medium of Periodontal Ligament-Derived Stem Cells on Chondrocytes, Synoviocytes, and Meniscus Cells.* Stem Cells Dev, 2021. **30**(10): p. 537-547.

2. Mustonen, A.M., et al., *Equine osteoarthritis modifies fatty acid signatures in synovial fluid and its extracellular vesicles.* Arthritis Res Ther, 2023. **25**(1): p. 39.

3. Cosenza, S., et al., *Mesenchymal stem cells derived exosomes and microparticles protect cartilage and bone from degradation in osteoarthritis.* Sci Rep, 2017. **7**(1): p. 16214.

4. Hotham, W.E., et al., *The anti-inflammatory effects of equine bone marrow stem cell-derived extracellular vesicles on autologous chondrocytes.* Vet Rec Open, 2021. **8**(1): p. e22.

5. Clarke, E.J., et al., *Optical photothermal infrared spectroscopy can differentiate equine osteoarthritic plasma extracellular vesicles from healthy controls.* Anal Methods, 2022. **14**(37): p. 3661-3670.

6. Ni, Z., et al., *The exosome-like vesicles from osteoarthritic chondrocyte enhanced mature IL-1beta production of macrophages and aggravated synovitis in osteoarthritis.* Cell Death Dis, 2019. **10**(7): p. 522.

7. Liu, X., et al., *Extracellular Vesicles Released From Articular Chondrocytes Play a Major Role in Cell-Cell Communication.* J Orthop Res, 2020. **38**(4): p. 731-739.

8. Zhang, S., et al., *MSC exosomes mediate cartilage repair by enhancing proliferation, attenuating apoptosis and modulating immune reactivity.* Biomaterials, 2018. **156**: p. 16-27.
