# Supplementary material for: Multi-Omic Temporal Landscape of Plasma and Synovial Fluid-Derived Extracellular Vesicles Using an Experimental Model of Equine Osteoarthritis
Source: Int J Mol Sci. 2023 Oct 4;24(19):14888. doi: 10.3390/ijms241914888 (PMC10573509; doi:10.3390/ijms241914888)

**Supplementary File 2.** Clustering analysis to determine the effect of missing values.

A. Cluster analysis of plasma-derived EVs, and B. SF-derived EVs, C. Density plot of plasma-derived EVs showing that when all the rows with missing values were removed the overall distribution shifted higher, D, Density plot of SF-derived EVs; the maximum number of proteins consistently identified in the samples was 50 and that was still in only 26 of the samples. Many proteins were identified in <10 of the samples, which may be indictive of the SF being lower quality SF samples might be generally variable.

1. Cluster plot for plasma-derived EVs


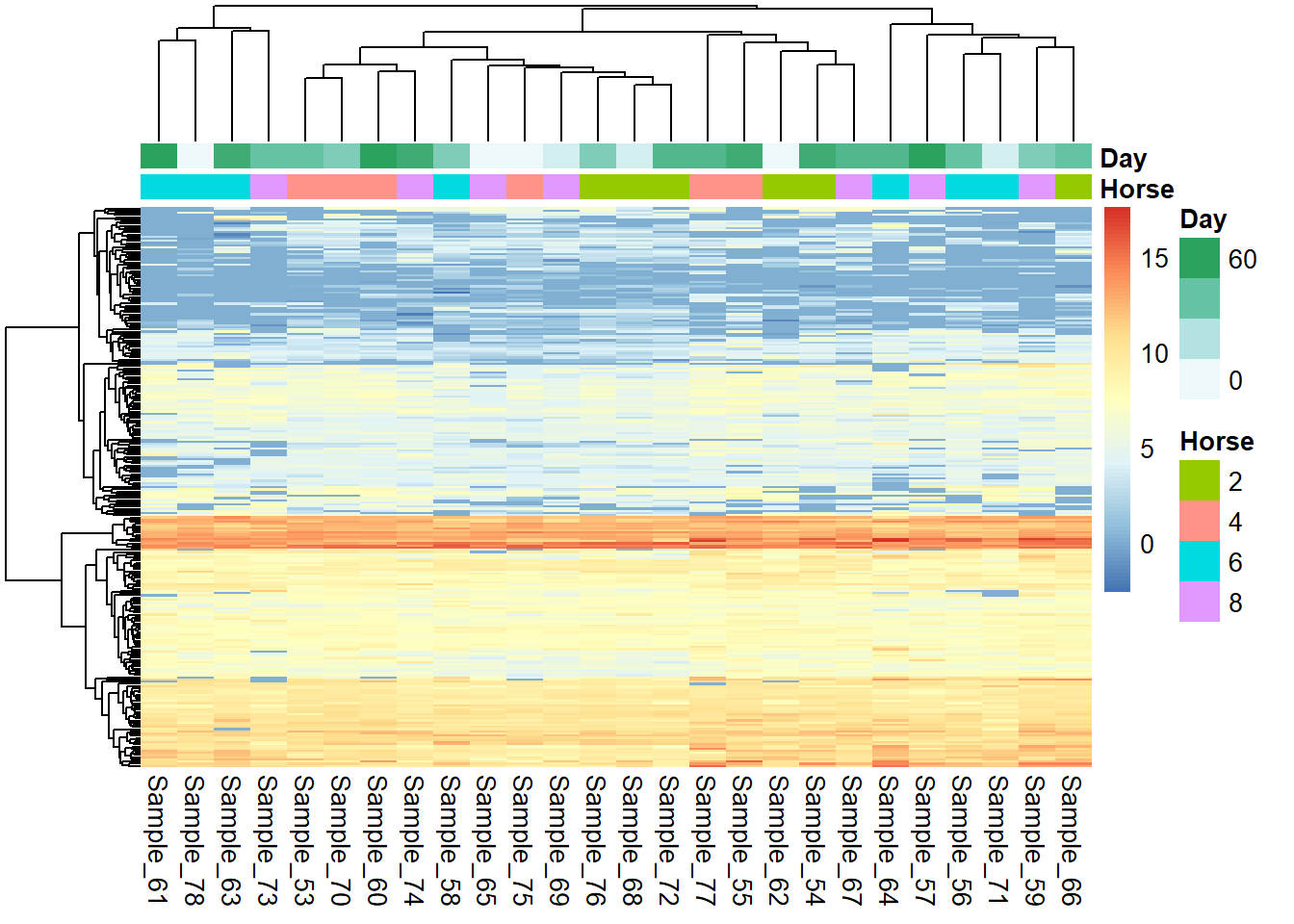


1. Cluster plot for SF-derived EVs


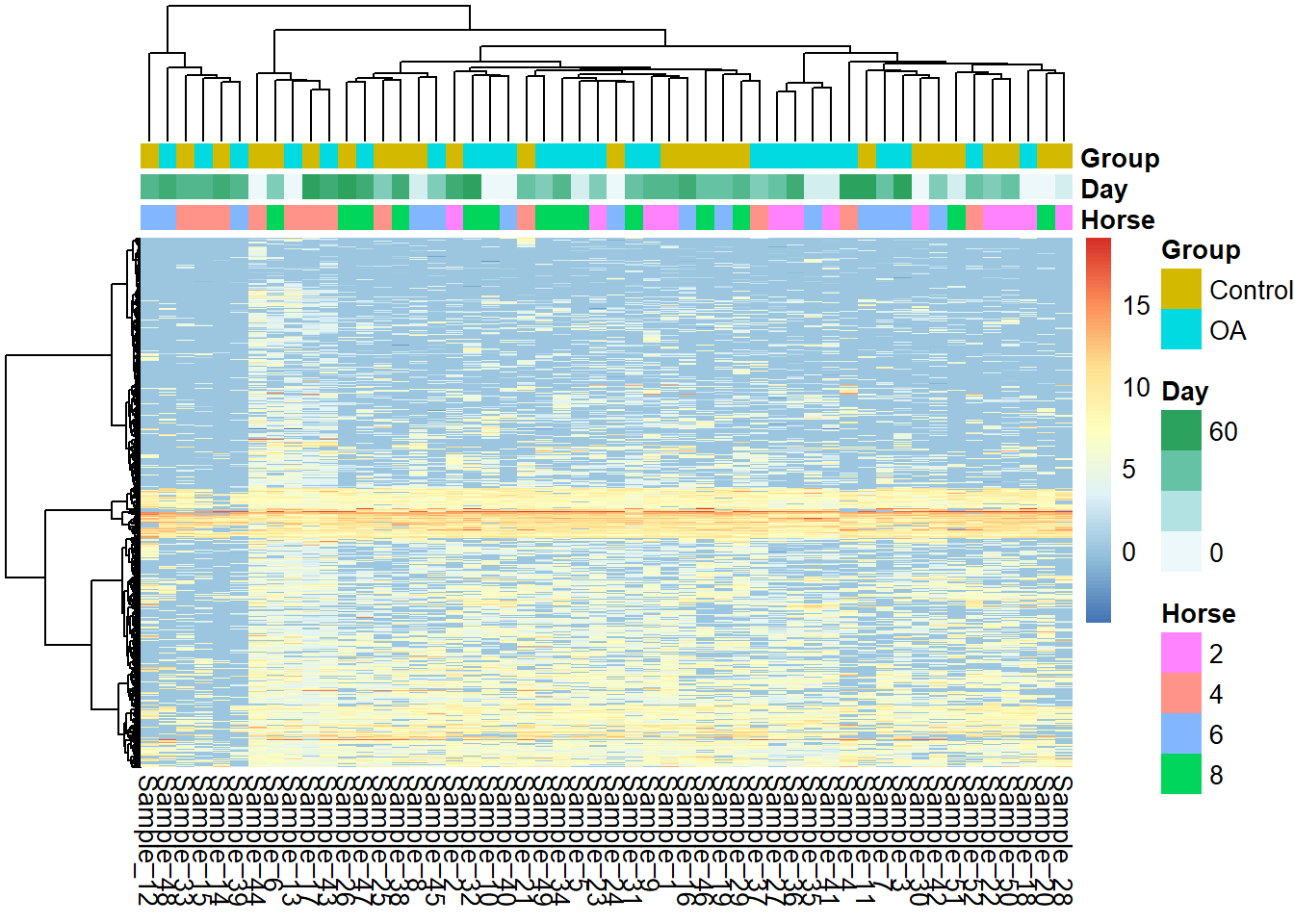


1. Plasma EV protein identification overlap plot. The plot shows the number of proteins identified across the samples. Most proteins were consistently identified across all 26 of the plasma samples.


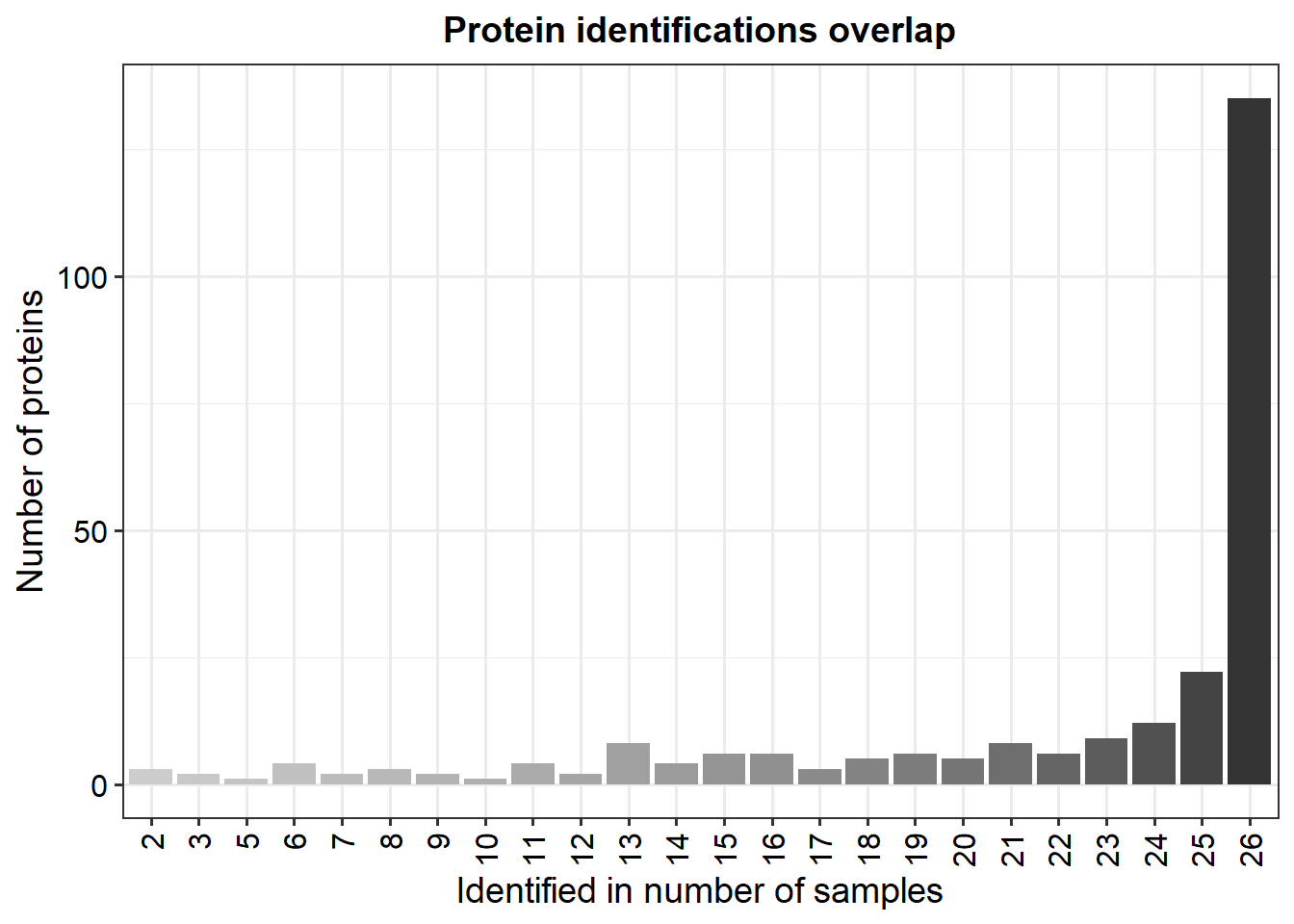


1. Synovial fluid EV protein identification overlap plot. The plot shows the number of proteins identified across the samples. For example, 52 proteins were identified in 26 samples. 30 proteins were identified in only 3 samples. Of the 52 synovial fluid samples no proteins were consistently identified across all samples. This is in contrast to the plasma EV samples which were much most consistent.


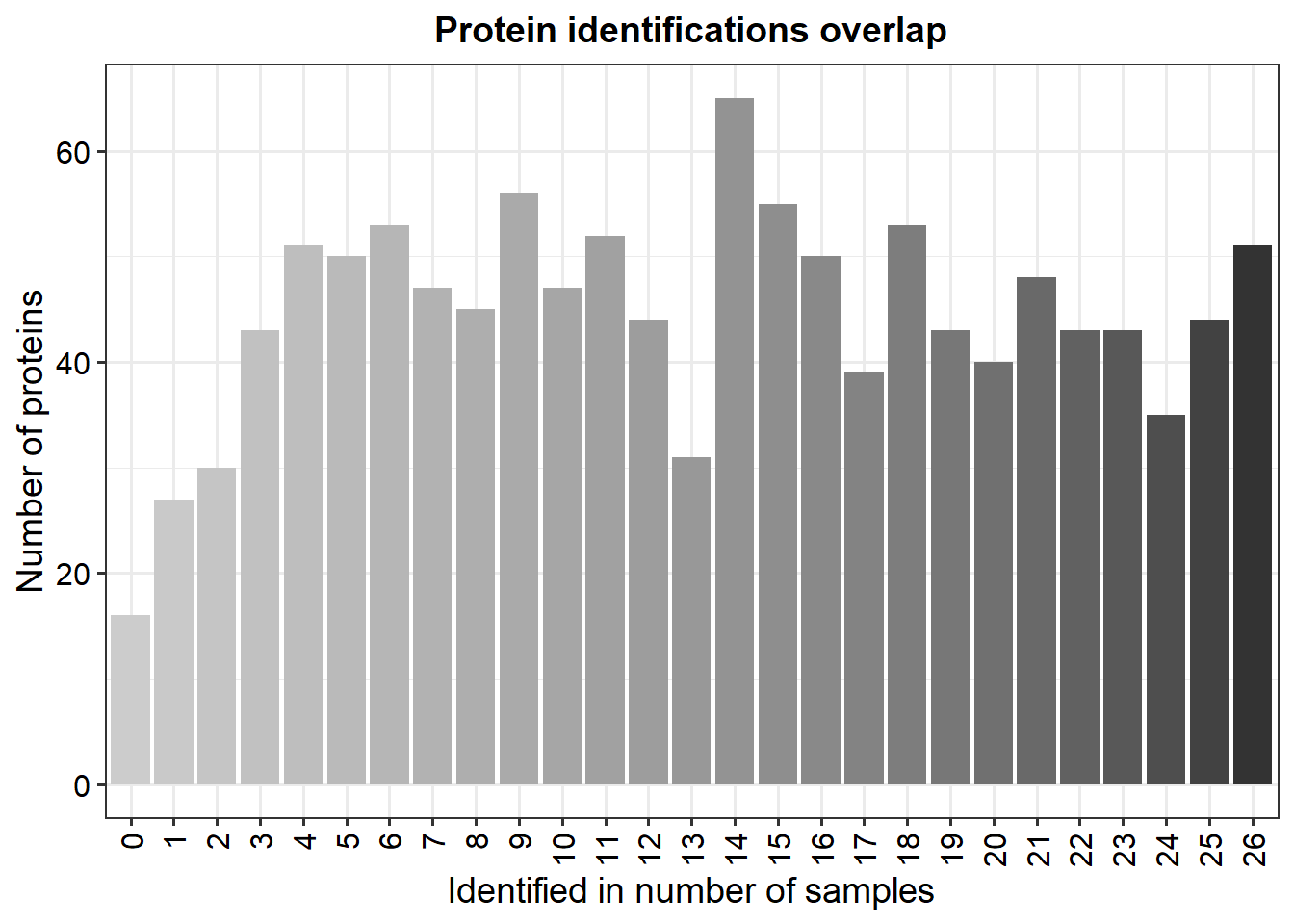

Supplement: Supplementary file 1 [file ijms-24-14888-s001.zip › Supplementary File 2 QC missing values _V2.docx]
