# Supplementary material for: Multi-Omic Temporal Landscape of Plasma and Synovial Fluid-Derived Extracellular Vesicles Using an Experimental Model of Equine Osteoarthritis
Source: Int J Mol Sci. 2023 Oct 4;24(19):14888. doi: 10.3390/ijms241914888 (PMC10573509; doi:10.3390/ijms241914888)

Supplementary File 5. Assessment of whether the missing values in small RNA sequencing data were true zeroes or sampling zeroes using PCA with complete observations compared to PCA with missing values as zeroes. A. Without missing valus, B. Missing values as zeros, C. Corelation map without missing values, D. Correlation map with missing values. When the missing values were included as 0’s PC1 had an almost 1:1 correlation with sequencing depth. Example shown is for miRNAs.


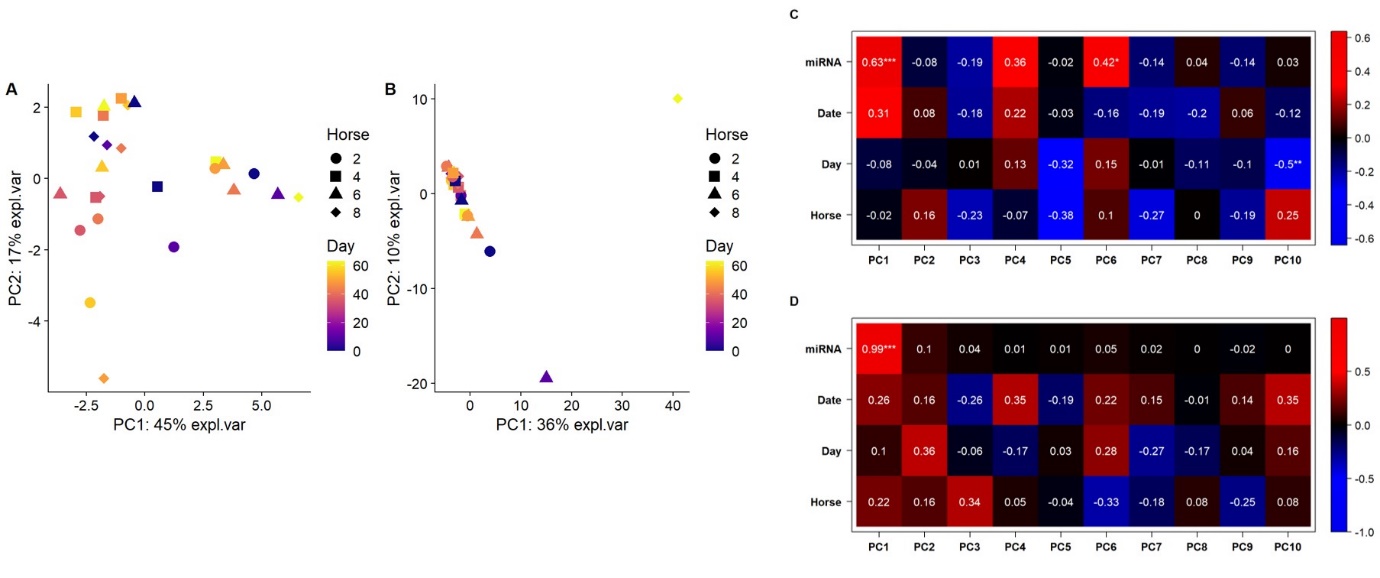

Supplement: Supplementary file 1 [file ijms-24-14888-s001.zip › Supplementary File 5 PCA for MV.docx]
